# Interaction of Brain-derived Neurotrophic Factor, Exercise, and Fear Extinction: Implications for Post-traumatic Stress Disorder

**DOI:** 10.2174/1570159X21666230724101321

**Published:** 2023-08-04

**Authors:** Emily J. Antolasic, Emily J. Jaehne, Maarten van den Buuse

**Affiliations:** 1 School of Psychology and Public Health, La Trobe University, Melbourne, Australia

**Keywords:** Post-traumatic stress disorder, anxiety disorders, brain-derived neurotrophic factor, exercise, Val66Met, stress

## Abstract

Brain-Derived Neurotrophic Factor (BDNF) plays an important role in brain development, neural plasticity, and learning and memory. The Val66Met single-nucleotide polymorphism is a common genetic variant that results in deficient activity-dependent release of BDNF. This polymorphism and its impact on fear conditioning and extinction, as well as on symptoms of post-traumatic stress disorder (PTSD), have been of increasing research interest over the last two decades. More recently, it has been demonstrated that regular physical activity may ameliorate impairments in fear extinction and alleviate symptoms in individuals with PTSD *via* an action on BDNF levels and that there are differential responses to exercise between the Val66Met genotypes. This narrative literature review first describes the theoretical underpinnings of the development and persistence of intrusive and hypervigilance symptoms commonly seen in PTSD and their treatment. It then discusses recent literature on the involvement of BDNF and the Val66Met polymorphism in fear conditioning and extinction and its involvement in PTSD diagnosis and severity. Finally, it investigates research on the impact of physical activity on BDNF secretion, the differences between the Val66Met genotypes, and the effect on fear extinction learning and memory and symptoms of PTSD.

## 1 INTRODUCTION

According to the World Mental Health survey, around 70% of the population has reported exposure to one or more traumatic events within their lifetime [1]. Although most individuals exposed to a traumatic event will recover, cross-national studies suggest that the lifetime prevalence of Post-Traumatic Stress Disorder (PTSD) is around 5% among trauma-exposed individuals and ranges from 0.5% to 14.5% across countries [1-4]. With respect to gender differences, the overall lifetime prevalence of PTSD is reported to be about 10-12% in women and 5-6% in men [5]. According to the Diagnostic and Statistical Manual of Mental Disorders, 5^th^ Edition (DSM-5), PTSD is characterised by the presence of intrusive recollections, hyperarousal and reactivity, persistent avoidance of stimuli, and negative alterations in cognition and mood following exposure to a traumatic event [6].

Various psychosocial and environmental risk factors have been shown to increase the risk of the development of PTSD, such as the severity of the trauma, younger age at the time of trauma exposure, lower socioeconomic status, lower level of education, negative coping strategies, and lack of social support [6]. Early molecular and twin studies have suggested that PTSD is moderately inheritable, although the specific genes implicated remain unclear [7, 8]. A more recent genome-wide association study (GWAS) in a large cohort of around 200,000 individuals concluded that genetic factors account for between 5 and 20% of the variability of PTSD risk, similar to the genetic component of other mental illnesses, such as major depression [9]. These factors could include genes involved in brain plasticity and resilience.

This narrative review will first briefly describe the theoretical underpinnings of the development and persistence of intrusive and hypervigilance symptoms commonly seen in PTSD, as these symptoms are the most well-researched within the literature and have been proposed to be mediated by neurobiological processes involved in learning and memory. It will then explore the current literature on the role of the neurotrophin, Brain-Derived Neurotropic Factor (BDNF), and its common single-nucleotide polymorphism, Val66Met, on fear extinction learning and memory in both human and animal models, including the relationship between this polymorphism and PTSD diagnosis and symptom severity. Finally, the feasibility of physical activity in treating impaired fear extinction and the potential role of increased BDNF expression will be analysed.

## 2 THEORIES AND STRUCTURES INVOLVED IN FEAR LEARNING AND MEMORY

The two-factor learning theory of fear and anxiety has been used to explain intrusive and hypervigilance symptoms seen in PTSD [10]. According to this theory, fear is acquired through classical conditioning processes and is maintained through operant conditioning through the negative reinforcement of avoidance behaviours [10]. Individuals with PTSD associate various stimuli present during the traumatic event with the memory of the event, causing these previously neutral stimuli to elicit fear and anxiety behaviors as conditioned responses [11]. In PTSD, these fear responses can become generalised to similar stimuli not present during the traumatic event, triggering flashbacks of the event and high levels of autonomic arousal. Individuals with PTSD will consequently avoid external and internal triggers to evade these feelings of anxiety and fear, which can include symptoms of panic. Exposure therapy utilises this theory to treat PTSD and other anxiety disorders by gradually exposing these individuals to stimuli associated with the traumatic event until they fail to elicit a fear response through extinction learning [11].

The Pavlovian classical conditioning paradigm has been utilised in animal models to study the acquisition and extinction of intrusive recollections and hyperreactivity symptoms of PTSD [12, 13]. This paradigm states that associative learning comprises the pairing of a neutral conditioned stimulus (CS) with an aversive unconditioned stimulus (US), which then elicits a conditioned fear response (CR) [14]. Fear extinction occurs when the CS is presented in the absence of the US, causing a gradual reduction in the magnitude and frequency of the CR [15]. However, in some individuals, the CR will reappear; for example, spontaneous recovery refers to the re-emergence of an extinguished CR after a period of extinction [16]. Renewal is when the CS is presented in a different context than the original CS-US pairing and elicits a CR. Reinstatement refers to the recovery of a CR after re-exposure to the US following extinction [14-16].

As could be expected, cerebral structures involved in contextual and cue-dependent fear regulation have also been implicated in PTSD, and it has been noted that the neural circuitry involved in fear and anxiety, also implicated in PTSD in humans, is highly conserved throughout evolution [12]. Brain regions involved include the hippocampus, a structure responsible for associative learning and pairing contextual information with memories, and the amygdala, which is responsible for storing fear memories and the expression of fear [17]. Two structures within the medial prefrontal cortex (mPFC), the dorsal anterior cingulate cortex (dACC), also known as the prelimbic cortex (PL) in rodents, and the ventromedial prefrontal cortex (vmPFC), also known as the infralimbic cortex (IL) in rodents, have been shown to have opposing influences on fear processing. The dACC is crucial for fear learning, while the vmPFC is required for fear extinction [17, 18].

In the brain, several neurotransmitter systems have been implicated in PTSD symptoms, notably norepinephrine, serotonin and glutamate, likely in conjunction with hypothalamic-pituitary-adrenal (HPA) axis dysfunction [19]. Not surprisingly, pharmacotherapy for PTSD may include drugs that target these systems, including selective serotonin reuptake inhibitors (SSRIs), selective serotonin and norepinephrine reuptake inhibitors (SNRIs), tricyclic antidepressants [20], and atypical antipsychotics, which target several of these neurotransmitters [19]. However, no single pharmacotherapy has been shown to be fully effective against PTSD symptoms, and only two are FDA-approved in the USA, the SSRIs sertraline and paroxetine. This may be caused by the condition showing substantial variability in symptom type and severity, as well as the possible role of genetic variants which influence the effectiveness of drug treatments [9]. This review will focus on one such source of genetic variability, neurotrophic adaptability in the brain, which has also been proposed as a target mediator of current pharmacotherapy, as well as alternative treatments, such as exercise.

## 3 BRAIN-DERIVED NEUROTROPHIC FACTOR

Neurotrophins comprise a family of proteins that play an essential role in synaptic function, plasticity and neuronal survival, and responses to stress within the mammalian nervous system [21, 22]. BDNF is a neurotrophin that is a prominent promoter of neuronal growth and plasticity in the brain throughout the lifespan [23]. The BDNF gene is located on chromosome 11p13-14 and comprises one main coding exon and multiple promotors to direct the site-specific transcription of the mature BDNF protein [24]. The BDNF protein is first synthesised as a 32 kDa precursor molecule called proBDNF, which itself can bind to the p75 receptor and the sortilin receptor complex to invoke apoptosis [25]. However, most proBDNF is cleaved intra- and extracellularly by plasmin, furin, and protein convertases into truncated or 14 kDa mature BDNF [24-26]. Until recently, the function of truncated BDNF was largely unknown but is now thought to be involved in altering neuronal physiology [27].

BDNF and its receptor, Tropomyosin-Related Kinase B (TrkB), are found within brain structures involved in learning and memory, such as the cortex, hypothalamus, hippocampus, and amygdala [28], each of which has also been implicated in anxiety disorders and PTSD. The binding of mature BDNF to TrkB triggers several signalling cascades that modulate synaptic transmission and plasticity [24, 29] and enhance neurogenesis and cell survival [30]. Such modulating effects of BDNF at the synapse have been deemed essential for molecular and electrophysiological processes required in long-term learning and memory [29-31], which could be involved in PTSD. Specifically, BDNF is secreted during the induction of long-term potentiation (LTP) and influences synaptic strength in a complex time- and brain region-dependent manner, as reviewed recently [32]. Enhanced LTP following TrkB activation, either by BDNF or other mechanisms, involves phospholipase Cγ and subsequently cyclic AMP-responsive element-binding protein (CREB) [29].

It should be noted that mice that are heterozygous for BDNF and consequently only have approximately half of the concentration of the neurotrophin in the brain [33] show intact spatial memory [34, 35], suggesting compensatory mechanisms to maintain optimal cellular signalling, for example, up-regulation of other neurotrophins, such as NT-4 [33]. In contrast, marked cognitive deficits have been described in reports on patients with BDNF heterozygosity [36] or mutations in the gene encoding for TrkB and NTRK2 [37, 38].

In terms of pharmacotherapy, there is reasonable consensus that SSRIs and other antidepressant drugs are associated with increases in BDNF levels in the periphery and brain [39, 40], providing a possible explanation for the beneficial role of the neurotrophin in anxiety disorders, such as PTSD. Of interest, more recent work has shown that both typical and fast-acting antidepressants also directly bind to TrkB [41], thereby facilitating its activation by BDNF. Either mechanism may be involved in the beneficial actions of SSRIs in PTSD.

## 4 BDNF AND FEAR CONDITIONING IN ANIMAL MODELS

Several animal studies have demonstrated the role of BDNF in consolidating fear memories and extinction. Rodents who received intracranial BDNF infusion showed differential responses based on the stage of the fear conditioning paradigm and the location into which BDNF was injected. With respect to the frontal cortex, infusion of BDNF in the IL, but not the PL, after fear conditioning resulted in fear extinction in rats in the short and long term [42, 43]. Rats that underwent a chronic fear conditioning paradigm showed increased BDNF expression in the prelimbic cortex compared to those who underwent fear extinction training [44]. Rosas-Vidal *et al.* [42] found no changes in BDNF expression in the IL and PL following extinction training, although expression was increased in the hippocampus. However, Kataoka *et al.* [43] found that BDNF was decreased in both the mPFC and hippocampus, and decreased TrkB phosphorylation was also seen in the vmPFC. Infusion of BDNF into the IL has also been shown to reduce conditioned fear for up to 48 hours, even in the absence of extinction training [45]. In this study, rats that demonstrated impaired fear extinction also showed reduced BDNF in hippocampal inputs to the IL [45]. Another study found higher BDNF expression in the mPFC and the amygdala and reduced expression in the IL and hippocampus following an inescapable foot shock paradigm [46].

With respect to the hippocampus, administration of recombinant BDNF into this brain region at retrieval has been shown to constrain fear memory extinction after prolonged memory recall [47]. Additionally, rats that received an intrahippocampal infusion of anti-BDNF antibodies immediately and six hours after fear extinction training exhibited impaired fear extinction memory [48]. Infusing these antibodies after extinction memory activation impeded the recovery of the avoidance response [48]. Chaaya *et al.* [49] demonstrated that contextual fear conditioning produced more microglia and cells expressing BDNF in the dentate gyrus than unpaired fear conditioning. These studies demonstrated the involvement of BDNF in brain structures involved in contextual learning and memory and its role in fear extinction learning, memory, and recovery.

Rodent models of deficient BDNF expression have similarly demonstrated impairments in fear extinction. Male BDNF heterozygous knock-out mice were found to have impaired fear extinction memory [50-52], and this deficit appeared to become more pronounced with age [50]. The deficits in fear extinction were accompanied by a reduction of BDNF in the hippocampus, amygdala, and PFC [52]. BDNF-e4 mutant mice demonstrated impaired extinction of fear compared to wild-type controls, with increased hippocampal activation and decreased hippocampal-mPFC activation early in the extinction training, as well as increased activation of the mPFC during extinction recall [53]. Additionally, selective neocortical BDNF knock-out mice demonstrated deficits in the consolidation of cued fear but not in the acquisition or expression of learned fear [54]. This deficit in cued fear consolidation was then rescued with TrkB agonists, indicating that BDNF-TrkB signalling plays an important role in this process [54] and that deficient BDNF signalling is associated with impaired cued fear consolidation and extinction. In addition to age, it is possible that cognitive deficits in BDNF heterozygosity are only seen in the presence of, or are exacerbated by, additional environmental factors, such as stress [34]. This would be in line with the well-accepted role of neurotrophins in general, and BDNF in particular, in neuroplasticity and resilience in response to environmental stress [22, 55].

**Fig. (1):**
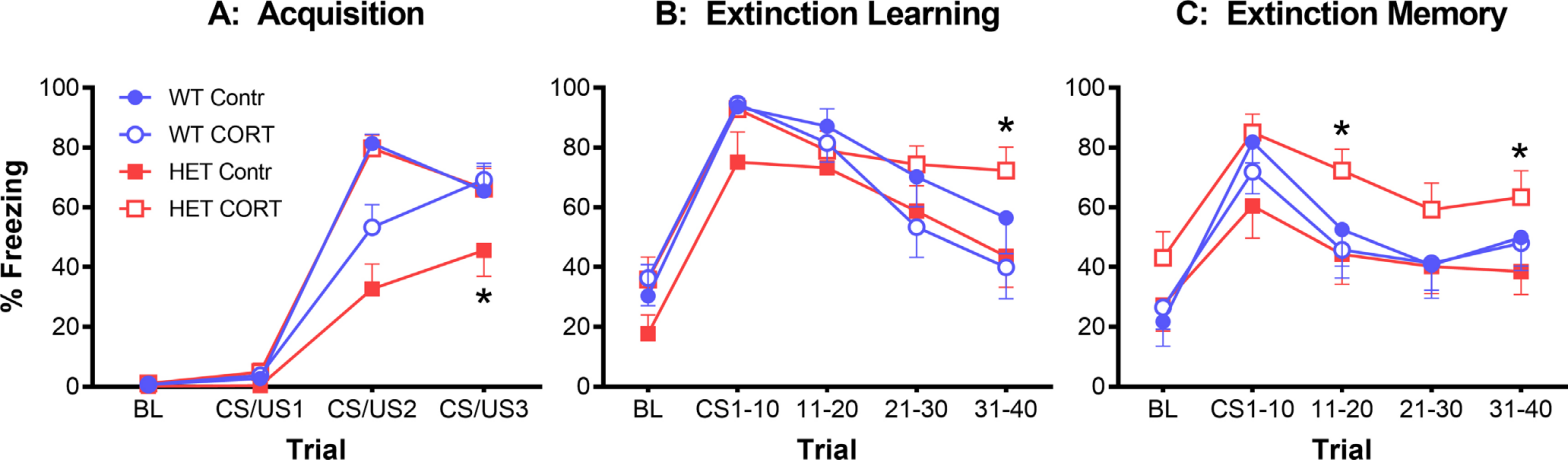
BDNF deficiency in BDNF heterozygous rats impairs fear acquisition (**A**) and leads to vulnerability to the effect of chronic exposure to the glucocorticoid stress hormone, corticosterone. BDNF heterozygous rats treated with corticosterone showed impaired fear extinction learning (**B**) and fear extinction memory (**C**). *p < 0.05 for difference in freezing compared to the corresponding wildtype group. **Abbreviations:** WT = wildtype control; HET = BDNF heterozygous; Contr = control treatment; CORT = corticosterone treatment in the drinking water. For methodological details and further results, see [56] from which this figure was adapted.

To study this gene-environment interaction, we used BDNF heterozygous rats and tested them in a standard three-day fear acquisition/extinction protocol [56]. Chronic stress was simulated by administrating the animals with the glucocorticoid and corticosterone in the drinking water of test animals. BDNF heterozygous rats showed reduced fear acquisition, and corticosterone treatment enhanced this to the level of wild-type rats (Fig. **1A**). Importantly, when the animals underwent a fear extinction phase where they were repeatedly exposed to the CS without the accompanying US, reduced tone-induced freezing was observed as expected in all rats, except BDNF heterozygous rats treated with corticosterone, in which the development of fear extinction was reduced (Fig. **1B**) and which showed impaired fear extinction memory on the final day of the protocol (Fig. **1C**) [56]. These results suggested that the role of BDNF in fear acquisition and extinction may be to increase vulnerability to external factors, including chronic stress.

Most of these studies only used male rodents; therefore, the results are not necessarily generalisable to both sexes. As females are more likely to develop PTSD and tend to have greater severity of symptoms [2-5], a sex-specific vulnerability is important to investigate. BDNF expression and effects of BDNF deficiency have been shown to be influenced by circulating sex steroid hormone levels [57]. Baker-Andresen *et al.* [58] found that female mice demonstrated deficits in the retention of fear extinction memory compared to male mice. They also found that naïve female mice exhibited more significant methylation and decreased BDNF exon IV mRNA levels than naïve males [58].

Although these studies support the involvement of BDNF in fear learning and memory and demonstrate the neural structures involved in these processes, they lack generalisation to human physiology. Some of the animal models show extreme genetic modifications that do not occur or are extremely rare naturally in humans [12, 36]. There are no imaging methods to study altered BDNF signalling in the human brain; therefore, clinical studies have largely relied on measurement of circulating levels of the neurotrophin.

## 5 SYSTEMIC BDNF AND PTSD

Human studies have demonstrated inconsistent evidence for the involvement of BDNF in PTSD. Aksu *et al.* [59] found that serum BDNF and proBDNF levels were significantly lower in individuals with a diagnosis of PTSD compared to controls but found no correlation with the severity of symptoms. Stratta *et al.* [60] found that patients who reported more PTSD symptoms had lower serum BDNF than patients who reported fewer symptoms and controls, suggesting some involvement of BDNF in the severity of symptoms in PTSD. Controversially, some studies have found an increase in serum BDNF levels in individuals with a diagnosis of PTSD in comparison to ‘healthy’ controls [61, 62]. Matsuoka *et al.* [62] also found a positive correlation between serum BDNF levels and the severity of symptoms over six months. However, other studies have not found any correlation among BDNF levels, PTSD diagnosis, and symptom severity. For example, Su *et al.* [63] found no significant differences in the rates of PTSD diagnosis in road traffic accident victims depending on their BDNF plasma levels 48 hours and six months after the accident. However, those without a diagnosis showed a trend where BDNF levels increased over time [63], suggesting BDNF is reactive to stress and secretion may increase over time as the initial impact of stressors lessens.

In addition to reduced serum levels of BDNF in PTSD, studies have identified altered epigenetic control of BDNF gene expression [64, 65]. For example, higher DNA methylation at four CpG sites of the BDNF gene promoter was found in veterans exposed to combat in the Vietnam war and with PTSD compared to those exposed to combat but without PTSD. High methylation levels at the BDNF promoter CpG site and high combat exposure were significantly associated with PTSD diagnosis [65].

Overall, it should be noted that some of the studies described here used relatively small sample sizes, lacked comparison to healthy controls, or gathered their samples from one institution; therefore, their results could be influenced by sample biases. Additionally, many of these studies are correlational, and there is little control over other psychosocial factors influencing the results; therefore, they cannot conclude that the results reflect only the influence of BDNF. It is also unclear whether changes in BDNF are a reaction to trauma or a premorbid vulnerability in these populations. As BDNF plays different roles in different brain regions, it is furthermore extremely difficult to reliably determine the direct relationship between BDNF levels and individual PTSD symptoms by measuring peripheral BDNF. PTSD is a complex disorder and likely has multiple psychobiological influences; therefore, it is possible that BDNF is not involved in all symptoms of PTSD.

The same problem arises when considering systemic BDNF-like treatments in a clinical setting. In humans, relatively little is known about interventions that impact BDNF signalling and may reduce PTSD symptoms. Some reports suggest that circulating levels of BDNF reflect changes in central levels; however, it is unclear if this applies to the transport of BDNF into the brain. Clearly, intra-cerebral injections are not feasible in humans, and systemically-injected BDNF is unlikely to reach relevant brain regions in effective concentrations unless, as yet, unapproved carrier mechanisms are employed [66]. Other strategies to increase BDNF signalling in the brain could include antidepressant drugs, some of which are known to increase BDNF expression [39, 40] or exercise.

Non-peptide TrkB receptor agonists are an alternative strategy to target central BDNF signalling [67], although this approach will still not be brain region-specific. Nevertheless, the pre-clinical and animal model literature shows promising results. Activation of TrkB by the flavone, 7,8-dihydroxy-flavone (7,8-DHF), reversed age-related deficits in contextual and cue-induced fear conditioning [68]. The authors suggested this effect of systemic administration of the flavone compound was mediated predominantly by an action in the amygdala [68]. A similar finding was reported by Andero *et al.* [69], who reported that 7,8-DHF enhanced both the acquisition of fear and its extinction, as well as rescued an extinction deficit caused by immobilization stress in mice [69]. Choi *et al.* [54] showed that the deficit in fear learning in mice with selective BDNF knockout in the prelimbic cortex could be reversed by systemic treatment with 7,8-DHF. Finally, specifically focusing on sex differences, impaired fear extinction in female mice compared to male mice could be rescued by systemic 7,8-DHF treatment [70]. Similar studies have not been done in humans, but the literature on animal models with 7,8-DHF supports the role of BDNF in fear conditioning and extinction and shows promise for the development of clinically-accepted drugs targeting BDNF signalling. Such an approach may need to take into account the effect of gene variants, particularly the BDNF Val66Met single-nucleotide polymorphism, which will be discussed in the next section.

## 6 BDNF VAL66MET POLYMORPHISM

Minor variants in a gene sequence can have a significant effect on gene expression and its regulation. A common genetic variation in the BDNF gene is the Val66Met polymorphism [23, 24]. This polymorphism involves a single-nucleotide substitution of guanine to adenine at position 196, resulting in an amino acid residue shift from valine (Val) to methionine (Met) within codon 66 of the BDNF prodomain [23, 24]. This means that the polymorphism does not result in an altered amino acid sequence of mature BDNF, which is the predominant form of BDNF in cellular signalling [71, 72]. Instead, the modification interferes with the binding of proBDNF to sortilin and consequently with intracellular trafficking, ultimately leading to reduced activity-dependent BDNF release [73, 74] and reduced BDNF-TrkB signalling [24]. Cultured hippocampal neurons have demonstrated an 18% decrease in BDNF secretion in neurons carrying one Met allele and a 29% decrease in neurons carrying two Met alleles [75, 76]. In human studies, the Met allele was associated with poorer episodic memory and abnormal hippocampal activation on fMRI [77]. In both human and rodent models, the Val/Val genotype represents a “wildtype,” while a Val/Met reflects heterozygosity and the Met/Met genotype homozygosity [27]. The Val66Met polymorphism is carried by 0.55% of Sub-Saharan Africans, 19.9% of Europeans, and 43.6% of Asians but is carried by up to 72% in certain populations [78]. Thus, unless specifically controlled for, studies on the role of BDNF in neurological functioning and psychiatric illness risk may produce different findings because of differential proportions of individuals with the Val/Val, Val/Met or Met/Met genotype depending on the ethnic distribution of participant populations [27, 78].

## 7 VAL66MET AND FEAR CONDITIONING IN ANIMAL MODELS

The research on the Val66Met polymorphism and fear extinction memory demonstrates a more explicit link between the involvement of BDNF in PTSD. The majority of the current literature suggests that the Met allele is associated with deficits in fear association learning or the persistence of fear memories over time when compared to the Val/Val genotype [79-83]. Dincheva *et al.* [79] found no differences between genotypes in Val66Met knock-in mice in contextual fear expression, but Met/Met mice did show a delayed expression of contextual fear over time. Giza *et al.* [81] highlighted this polymorphism's effect at a neuronal level, as they found ventral CA1 hippocampal neurons in Met/Met mice fail to adapt their activity during fear extinction training, resulting in deficits in the persistence of fear memories. Soliman *et al.* [83] found impaired fear conditioning in those with the Met allele compared to the Val/Val genotype in both human and animal models. Additionally, they found atypical frontoamygdala activity in humans with the Met allele [83]. This provides evidence for the involvement of this polymorphism in the neural processes involved in fear association learning [83]. Similarly, Mühlberger *et al.* [82] found that Met-carrying individuals demonstrated a generalisation of fear responses in novel contexts, suggesting deficits in fear association learning. Others found heightened amygdala responses and reduced subgenual anterior cingulate responses in Met allele carriers during fear conditioning [84]. They also found heightened responses in the insula, amygdala, and hippocampus during early extinction in Met allele carriers but no differences during late extinction [84].

In marked contrast, some studies have suggested a protective effect of the Met allele as demonstrated by superior fear extinction learning and reduced fear memory and spontaneous recovery [85-87]. Jaehne *et al.* [87] suggested that the impact of this polymorphism seems specific to fear memory as no genotype differences were seen in measures of anxiety-like behaviour, such as on the elevated plus maze, or other memory measures, such as novel-object recognition or short-term spatial memory in the Y-maze. However, perhaps this polymorphism alone does not produce deficits in fear learning, extinction learning, and memory and, as discussed above, may interact with stress to increase vulnerability [88]. Animal studies have demonstrated promising evidence of such a genotype and stress interaction effect, as shown by impaired fear extinction in Met/Met mice exposed to chronic corticosterone treatment [89, 90]. Notaras *et al.* [90] also found an increase in the expression of glucocorticoid receptors in the dorsal hippocampus in this genotype during adolescence. Raju *et al.* [89] found a sex-specific interaction effect whereby corticosterone treatment selectively abolished fear extinction in female Met/Met mice, which also showed decreased amygdala GABAergic interneuron expression.

**Fig. (2):**
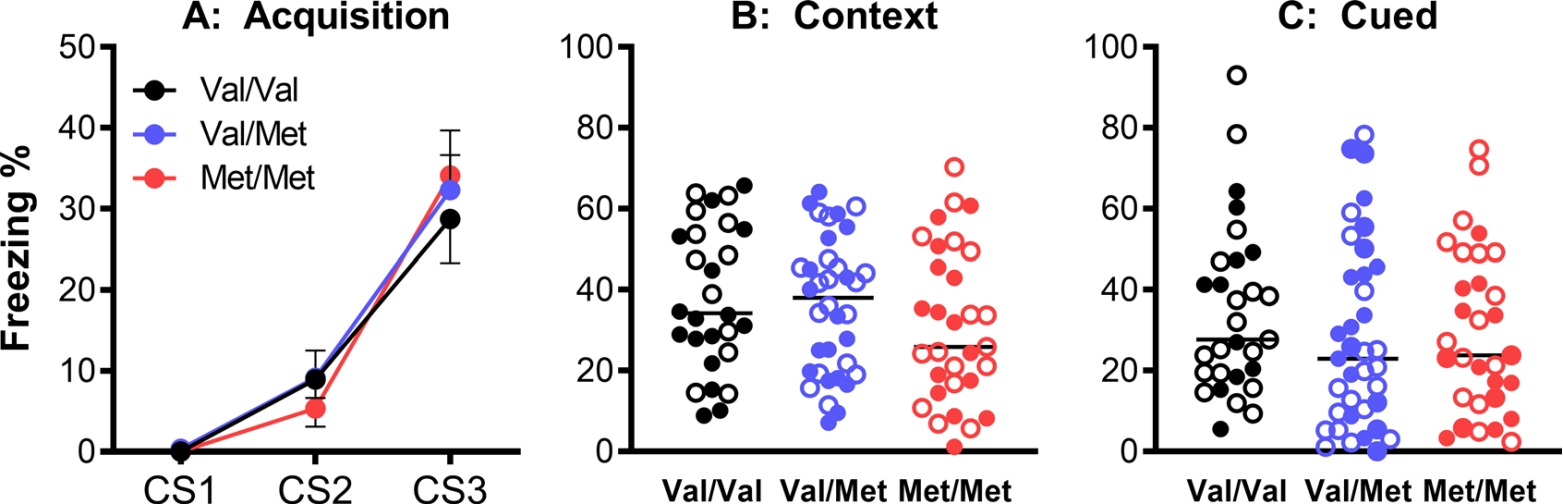
Lack of effect of the BDNF Val66Met genotype on acquisition of conditioned fear (**A**), and context- and cue-induced freezing (**B** and **C**), open symbols are data from females, closed symbols are data from males). Of note, these animals were part of a study which used a chronic treatment protocol including daily intraperitoneal injections, which may have constituted additional stress and influenced underlying genotype differences. For methodological details and further results, see [91] from which this figure was adapted.

Following earlier studies in BDNF heterozygous rats and Val66Met mice [56, 89, 90], we recently found no differences between Val/Val, Val/Met and Met/Met mice in terms of fear acquisition, contextual fear and cue-induced fear (Figs. **2A**-**C**). Interestingly, these mice were the vehicle-treated controls in a study in which the effects of a chronic methamphetamine treatment paradigm were investigated, including daily intraperitoneal injections [91]. It is possible that even in the vehicle-treated control mice in this study, the added chronic stress of repeated injections diminished underlying genotype differences, similar to the more controlled stress simulation by corticosterone treatment in other studies [90]. Therefore, it is important in animal model studies to take into account additional factors, such as housing conditions and frequency of handling, which could explain some of the discrepancies between study results [12, 92]. Other factors of relevance in the animal model literature are species differences [92, 93], rat and mouse strain differences [94] and, as mentioned above, sex differences [5, 95].

## 8 VAL66MET AND PTSD

The Val66Met polymorphism has been associated with PTSD diagnosis and symptom severity, although there are some inconsistencies between findings. Studies have found that the Met allele is overrepresented in PTSD populations compared to the Val/Val genotype [96-101]. The Met allele has also been associated with higher PTSD symptom severity than the Val/Val genotype [80, 96, 102, 103]. In addition, Young *et al.* [98] found that PTSD Met allele carriers demonstrated greater physiological responses in no threat and ambiguous threat conditions in a fear-potentiated acoustic startle paradigm, suggesting deficits in cued fear association learning. Felmingham *et al.* [80] found that poor fear conditioning was associated with greater PTSD symptom severity in individuals carrying the Met allele. Felmingham *et al.* [104] found that individuals with the Met allele with a diagnosis of PTSD demonstrated poorer responses to exposure therapy compared to patients with the Val/Val genotype. Similarly, Lyoo *et al.* [105] found that in individuals with a diagnosis of PTSD, the Val/Val genotype was associated with better symptom recovery than Met allele carriers.

In contrast, Jin *et al.* [106] found that the Val/Val genotype was associated with higher levels of childhood trauma and greater severity of PTSD symptoms in the Korean population. Additionally, a significant interaction in cortical thickness of the left fusiform and transverse temporal gyri and symptom severity in the Val/Val genotype was found [106]. However, some studies have found no association between the Val66Met genotype, PTSD diagnosis, and symptom severity [107-112], although Bruenig *et al.* [109] showed a trend towards a potential protective effect in the Val/Val genotype. Van den Heuvel *et al.* [107] also found that plasma BDNF was correlated with the number of lifetime trauma exposures, demonstrating BDNF’s reactivity to traumatic stress.

It is important to note that the vast majority of previous research has been conducted on male US veterans or military personnel or lacks comparison to a ‘healthy’ community population. Therefore, it lacks generalisability and may be influenced by sample biases. Another limitation of this research is that it relied on self-report for indications of past trauma, which is known to be subject to reporter biases. Furthermore, with many of the studies being correlational, there is no control over extraneous variables that may be influencing the results. Therefore, it is difficult to determine a cause-and-effect relationship between this polymorphism and PTSD susceptibility. However, it is unlikely that this polymorphism would increase vulnerability to PTSD on its own, and it likely interacts with stress, trauma exposure, and other genetic variations to increase susceptibility. Many of the human studies did not control for the current level of stress, which could have had an impact on learning and memory, and/or were based on highly educated Caucasian populations, therefore lacking generalisability. Additionally, most human studies had small sample sizes and uneven distributions between the genotypes, which might have affected the validity of the results. Overall, given this large number of limitations and potentially confounding factors, it is not surprising that a meta-analysis failed to find a genetic association between BDNF Val66Met and PTSD [111].

## 9 BDNF AND PHYSICAL ACTIVITY

Physical activity has been shown to provide cognitive benefits and regulate mood by stimulating increased BDNF concentrations in various brain regions [113, 114] and improving synaptic plasticity and cognition [115]. Animal studies have indicated that exercise increases BDNF concentrations in brain regions responsible for learning, memory, and emotional processing (*i.e*., the hippocampus, prefrontal cortex and amygdala) [116-119]. For example, an animal study using an inhibitory avoidance paradigm found that moderate exercise alleviated impaired fear extinction while increasing hippocampal BDNF [120]. In rats, voluntary exercise has similarly been shown to increase BDNF mRNA production in the hippocampus and neocortex, indicating that exercise increases the secretion of BDNF through the upregulation of BDNF gene transcription [121]. The exercise-induced increase in BDNF expression in the brain was associated with enhanced neurogenesis, including proliferation and neural differentiation of neural stem cells [122, 123] and may play a central role in stress resilience and PTSD-like behavior [124]. Indeed, treatment with the neurogenesis enhancer, memantine, improved social avoidance behavior in a PTSD model in rats [125]. Conversely, treatment with the neurogenesis inhibitor, valganciclovir, prevented long-term recovery of anxiety-like behavior and acute stress-induced corticosterone responses following a single combined stress model in rats [126].

Human studies have also demonstrated exercise-induced increased BDNF levels and responses to fear extinction training or exposure therapy in individuals with PTSD [127, 128]. This suggests the efficacy of using physical activity in addition to exposure-like therapies for the treatment of symptoms of PTSD. Meta-analyses have found an increase in peripheral blood BDNF levels after exercise in human participants, which was shown to increase with session duration [113, 129]. Regular exercise also intensified the increase in BDNF after a session [129]. These studies also found that women showed less change in their BDNF levels after exercise compared to males, suggesting that women may be less reactive to exercise-induced changes in BDNF secretion [113, 129]. Studies have shown increases in BDNF following exercise in other clinical disorders as well, such as depression, anxiety, neurodegenerative disorders and epilepsy, and have demonstrated benefits in terms of mood and cognitive functioning [130-133].

Differences in body composition and muscle mass may likely account for sex-specific differences in BDNF secretion. Increases in BDNF mRNA and BDNF expression have been observed in rats in response to muscle contraction [113], thus demonstrating that BDNF may be produced in the muscle during exercise. Therefore, as males generally have higher muscle mass than females, this may account for the greater production of BDNF. In addition, smaller samples of females compared to male participants may have resulted in the lack of significant findings among female participants. Additionally, only a few human studies have been able to demonstrate whether BDNF can cross the blood-brain barrier [113]. Although animal studies have demonstrated good evidence in support of this, it is, therefore, difficult to determine whether increases in peripheral BDNF indicate an increase in BDNF in the human cerebral cortex.

## 10 VAL66MET AND PHYSICAL ACTIVITY

**Fig. (3):**
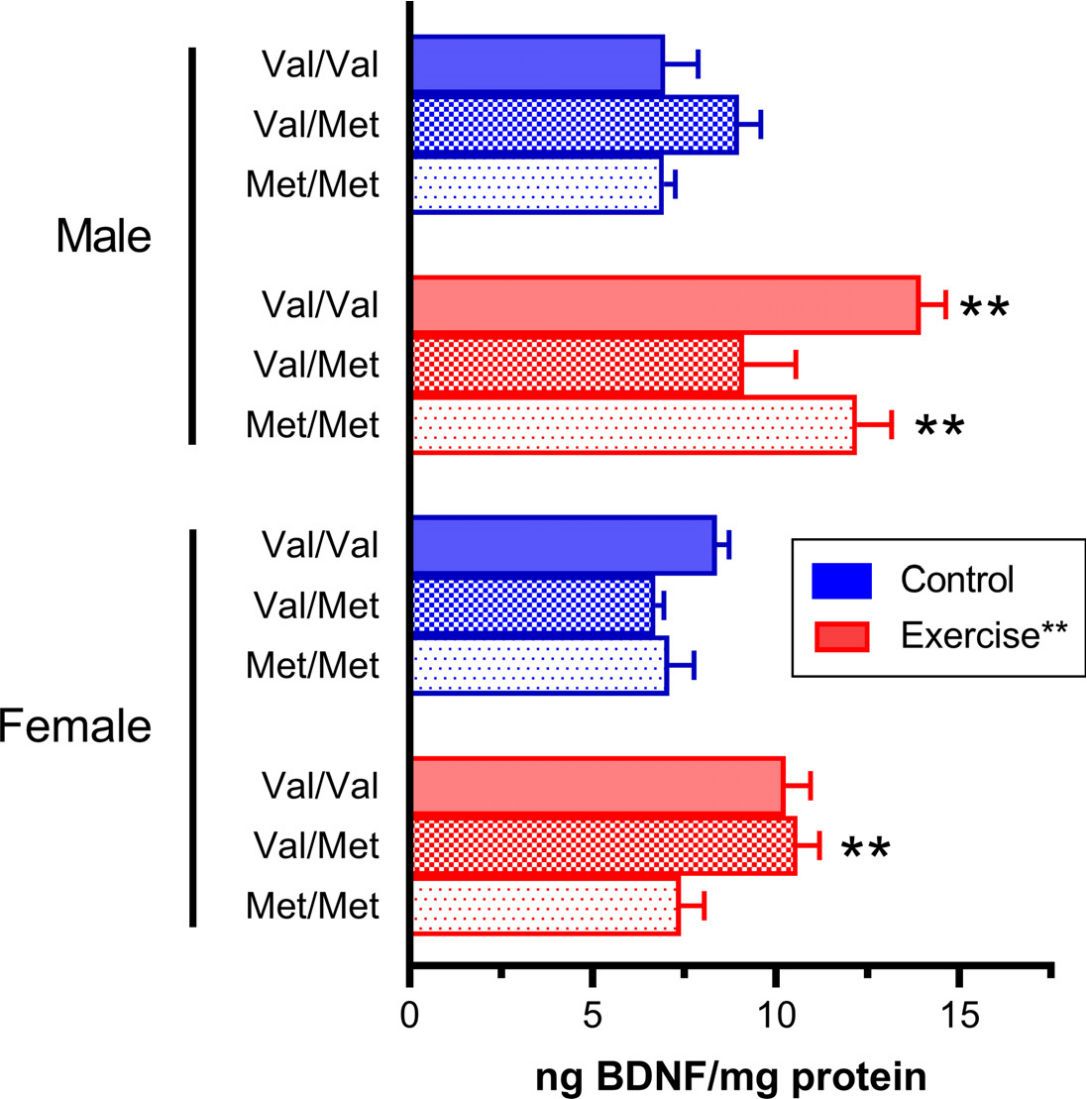
BDNF protein levels, measured with ELISA, are increased in the ventral hippocampus of a BDNF Val66Met model following chronic exercise. This effect was most pronounced in Val/Val and Met/Met male rats and in Val/Met female rats. Data are mean ± SEM. **p < 0.05 for difference with sedentary controls of the same sex and genotype. For methodological details and additional results, see [134], from which this figure was adapted.

There may be different responses among Val66Met genotypes following exercise. We recently confirmed this finding in a cohort of Val68Met rats that were housed in running-wheel cages and which developed high levels of exercise [134]. Exercise rats showed significantly higher BDNF gene expression in the hippocampus and levels of BDNF protein in the ventral hippocampus compared to sedentary rats housed under standard conditions [134], although some genotype differences were found between male and female rats (Fig. **3**). Specifically, the effect of exercise on BDNF protein levels was most pronounced in Val/Val and Met/Met male rats and in Val/Met female rats.

Clinically, some studies have indicated that those with the Met allele demonstrate significant improvements in BDNF levels post-exercise interventions compared to Val homozygotes [135, 136]. Other studies have demonstrated improved cognitive and mood outcomes in response to physical activity in Met carriers compared to those with the Val/Val genotype [137-140]. There appears to be some evidence for this at a neuronal level as the Met allele has been associated with increases in mean diffusivity in significant clusters throughout the bilateral hemispheres in response to exercise, despite at baseline having reduced mean diffusivity throughout the grey and white matter in comparison to the Val/Val genotype [141]. Caldwell *et al.* [142] found that those with at least one copy of the Met allele reported greater motivation to continue exercising and less perceived exertion than those with the Val/Val genotype. This suggests that those with the Met allele may perceive exercise as more intrinsically rewarding and, therefore, may be more likely to engage in higher levels of physical activity than those with the Val/Val genotype [142]. However, some studies have found an increase in BDNF levels after exercising in the Val/Val genotype, which was not seen in Met allele carriers [143, 144]. Specifically, Chen *et al.* [143] found a decrease in anxiety and depressive-like behaviour in mice carrying the Met allele after physical activity compared to mice with the Val/Val genotype. Additionally, Watts *et al.* [145] found that exercise benefited cognition only in males of the Val/Val genotype. However, some studies have found no association between the Val66Met genotype and BDNF increases after exercise [146].

Overall, this research has a good distribution across age groups and sexes/genders, making the results more generalisable. However, further research comparing the difference between exercise durations, intensity and type is needed to indicate what type of exercise provides the most benefit and whether this differs between the genotypes. Moreover, a large proportion of the current literature on this topic focused on populations who were cognitively compromised (*e.g*., mild cognitive impairment or had a diagnosis of a neurodegenerative disorder) and therefore lacked generalisation to healthy populations.

## 11 VAL66MET, PTSD, FEAR CONDITIONING, AND PHYSICAL ACTIVITY

A limited amount of research has been conducted on the effect of the Val66Met polymorphism on fear conditioning or symptoms of PTSD and the moderative effects of physical activity. Some studies suggest that individuals with the Met allele benefit most from exercise in fear extinction recovery [147, 148]. In contrast, others suggest that those with the Val/Val genotype are more likely to show benefits compared to Met allele carriers [149]. Keyan and Bryant [147] found that exercise was associated with greater fear recovery only in those with the Met allele in a potentiated startle paradigm. Pitts *et al.* [148] found that Met allele carriers reported greater severity of lifetime and current symptoms of PTSD, specifically re-experiencing symptoms. They also found that Met allele carriers who had exercised had significantly lower severity of PTSD symptoms than those who had not; however, the same effect was not seen in those with the Val/Val genotype [148]. However, Keyan and Bryant [149] found an interaction between the Val/Val allele and cortisol response, which predicted stronger emotional memory in the exercise condition, demonstrating that the Val/Val genotype may have a greater response from exercise in their memory of emotional information. Therefore, the interaction between the Val66Met polymorphism, physical activity, fear conditioning, and extinction remains unclear.

However, the majority of these studies utilised small sample sizes or focused on different aspects of symptomology. The studies conducted by Keyan and Bryant [147, 149] focused on the impact of acute exercise following a conditioning and extinction paradigm. In contrast, Pitts investigated the correlation between PTSD symptoms and self-reported current engagement in physical activity. Additionally, the studies by Keyan and Bryant [149] and Pitts *et al.* [148] used subjective measures, such as self-reports, to analyse symptoms of PTSD, memory and level of engagement in physical activity. Studies using standardised laboratory-controlled assessment measures are needed to reduce the influence of extraneous variables on the results. Therefore, further research is needed on the direct impact of regular exercise and this polymorphism on fear conditioning and extinction.

## CONCLUSION AND FUTURE DIRECTIONS

BDNF and its single-nucleotide polymorphism Val66Met have been associated with fear conditioning responses and PTSD susceptibility and symptom severity. There is some evidence to suggest a sex-specific genotype interaction, although studies analysing differences between the sexes are limited. This polymorphism has demonstrated differing effects at a structural, neuronal, cellular, and molecular level. Exercise has been shown to increase BDNF levels and improve mood, cognition and response to fear conditioning and extinction training. There is some evidence to suggest differing responses between the genotypes regarding their response to physical activity. However, small sample sizes, sampling biases, uncontrolled extraneous variables and methodological limitations may contribute to inconsistencies within the results across studies. Although the majority of the research appears to suggest that the Met allele is associated with impaired fear conditioning, higher susceptibility to PTSD, and a greater response to physical activity when assessing mood, cognition, and fear conditioning responses, there is increasing evidence to suggest that the Val/Val genotype may increase vulnerability and be more reactive to exercise-induced changes in BDNF levels.

Future research should investigate a sex-specific genotype interaction in response to physical activity in fear conditioning and extinction. Differences in the frequency and timing of exercise should also be explored (*i.e*., acute *vs*. regular exercise and exercise following fear learning *vs*. fear extinction training). Furthermore, the influence of a gene-environment interaction should be examined further.

## LIST OF ABBREVIATIONS

7,8-DHF: 7,8-Dihydroxyflavone
BDNF: Brain-derived Neurotrophic Factor
CR: Conditioned Fear Response
CS: Conditioned Stimulus
CREB: Cyclic AMP-responsive Element-binding Protein
DACC: Dorsal Anterior Cingulate Cortex
DSM-5: Diagnostic and Statistical Manual of Mental Disorders, 5^th^ Edition
GWAS: Genome-wide Association Study
HPA: Hypothalamic-pituitary-adrenal
IL: Infralimbic Cortex
LTP: Long-term Potentiation
Met: Methionine
mPFC: Medial Prefrontal Cortex
PL: Prelimbic Cortex
PTSD: Post-traumatic Stress Disorder
SNRI: Selective Serotonin and Norepinephrine Reuptake Inhibitors
SSRI: Selective Serotonin Reuptake Inhibitors
TrkB: Tropomyosin-Related Kinase B
US: Unconditioned Stimulus
Val: Valine
vmPFC: Ventromedial Prefrontal Cortex
